# Synergistic use of anti-inflammatory ketorolac and gentamicin to target staphylococcal biofilms

**DOI:** 10.21203/rs.3.rs-3471646/v1

**Published:** 2023-10-26

**Authors:** Amita Sekar, Dmitry Gil, Peyton Anne Tierney, Madeline McCanne, Vikram Daesety, Darina Trendafilova, Orhun K Muratoglu, Ebru Oral

**Affiliations:** 1 Harris Orthopaedic Laboratory, Massachusetts General Hospital; Boston, U.S.A.; 2 Department of Orthopaedic Surgery, Harvard Medical School, Harvard University; Boston, U.S.A

## Abstract

**Background:**

While antibiotics remain our primary tools against microbial infection, increasing antibiotic resistance (inherent and acquired) is a major detriment to their efficacy. A practical approach to maintaining or reversing the efficacy of antibiotics is the use of other commonly used therapeutics, which show synergistic antibacterial action with antibiotics. Here, we investigated the extent of antibacterial synergy between the antibiotic gentamicin and the anti-inflammatory ketorolac regarding the dynamics of biofilm growth, the rate of acquired resistance, and the possible mechanism of synergy.

**Methods:**

Control (ATCC 12600, ATCC 35984) and clinical strains (L1101, L1116) of *S. aureus* and *S. epidermidis* with varying antibiotic susceptibility profiles were used in this study to simulate implant-material associated low-risk and high-risk biofilms *in vitro*. The synergistic action of gentamicin sulfate (GS) and ketorolac tromethamine (KT), against planktonic staphylococcal strains were determined using the fractional inhibitory concentration measurement assay. Nascent (6hr) and established (24hr) biofilms were grown on 316 stainless steel plates and the synergistic biofilm eradication activity was determined and characterized using adherent bacteria count, MBEC measurement for GS, gene expression of biofilm-associated genes, visualization by live/dead imaging, scanning electron microscopy, and bacterial membrane fluidity assessment.

**Results:**

Gentamicin-ketorolac combination demonstrated synergistic antibacterial action against planktonic Staphylococci. Control and clinical strains showed distinct biofilm growth dynamics and an increase in biofilm maturity was shown to confer further resistance to gentamicin for both ‘low-risk’ and ‘high-risk’ biofilms. The addition of ketorolac enhanced the antibiofilm activity of gentamicin against acquired resistance in staphylococcal biofilms. Mechanistic studies revealed that the synergistic action of gentamicin-ketorolac interferes with biofilm morphology and subverts bacterial stress response altering bacterial physiology, membrane dynamics, and biofilm properties.

**Conclusion:**

The results of this study have a significant impact on the local administration of antibiotics and other therapeutic agents commonly used in the prevention and treatment of orthopaedic infections. Further, these results warrant the study of synergy for the concurrent or sequential administration of non-antibiotic drugs for antimicrobial effect.

## INTRODUCTION

Orthopaedic infections are a heavy healthcare burden(1) because they are unpredictable and difficult to definitively diagnose, and their treatment is often lengthy and complex comprising multiple surgeries and medication regimens(2,3). Nevertheless, the recurrence rate is 20–35% and is associated with dire consequences such as arthrodesis(4,5). The 5-year survival rates of periprosthetic infection (PJI; ~70%) are like those of some cancers(6,7). Thus, PJI is a serious condition with high morbidity and mortality for patients that requires immediate attention. Most periprosthetic infections occur within 2 years of the index surgery. ‘Acute’ infections present with obvious signs such as swelling, or a sinus tract. In ‘chronic’ cases, the clinical manifestation is less clear where the patient may have some pain or discomfort without overt signs. The current consensus is focused on using thresholds for systemic markers such as C-reactive protein and combining the results of blood tests such as that for leukocyte esterase to guide diagnosis and treatment decisions(8,9). The gold-standard approach is two-stage revision surgery which removes all implants followed by a period with intravenous (IV) antibiotics and an antibiotic-eluting bone cement spacer protecting the joint space in the meantime. When the infection is cleared [after about 4 months (10)], new devices are implanted. The cure rate of this approach is 54–77%(11).

Identification of bacteria, both in acute and chronic infections, is only possible by culturing intra-articular swabs. Treatment steps (medication and surgery) are often taken without knowing the details of the infecting bacteria(12) and there is a high probability of negative cultures (~20%)(13). Despite these shortcomings of identification, the majority of positive cultures comprise gram (+) microorganisms *Staphylococcus aureus (SA)* and *Staphylococcus epidermidis* (*SE*) (60–70%(14)). In infected revisions, ~50% of Staphylococcal infections are methicillin-resistant(15). Furthermore, coagulase-negative staphylococci (*S. epidermidis*) are known to persist due to their higher ability to form biofilms and are isolated from chronic infections more than other Staphylococcal species(16). Thus, Staphylococcus organisms are the most relevant for the control of PJI and have varied characteristics.

Implant devices serve as substrates for biofilm attachment and colonization. The long-held view of the importance of initial bacterial attachment to medical device surfaces is based on work showing that these avascular surfaces can provide a safe harbor for bacteria to evolve into a state of biofilm that acquires resistance to soluble drugs (17–19). Following adhesion, the biofilm is gradually established, with several subpopulations harboring varying phenotypic and genotypic signatures contributing significantly to the maturity and drug resistance(20). Thus, it is believed that the rate of acquired resistance due to the formation of aggregates can be slowed down or eradicated by preventing the adhesion of bacteria to surfaces(21,22).

The risk of treatment failure is a compound risk associated with the presence of staphylococcal infection, duration of the infection, host factors such as antecedent antibiotic exposure, immunocompetency, and the details of the treatment(23–25). Current prevention strategy incorporating antibiotic therapy involves MRSA screening of patients and peri-surgical systemic antibiotics(26–28). The treatment generally involves systemic broad-coverage antibiotic therapy together with revision surgery to remove biofilm. Combination therapy utilizing beta-lactams/cell wall inhibiting antibiotics together with biofilm-targeting drugs such as rifampicin has proven the most effective current strategy in eradicating implant and tissue-associated biofilms(27). Although inherent resistance to antibiotics is one of the major drivers of treatment outcomes, the implication of acquired resistance conferred by biofilm formation also warrants our attention. Antibiotics should be at an effective concentration at the site of infection for a significant period and they should be able to act on mature biofilms and persisters(29). Effective clearance of infections becomes a growing challenge for antibiotics due to the inaccessibility of drug targets and emerging resistant bacterial subpopulations (persisters) due to the prolonged presence of drugs. This increases the burden on antibiotics and the immune system to access deep tissue spaces to target the bacteria. Despite the challenges, antibiotics remain the mainstay of effective treatment, and increasing treatment success can be most likely obtained by targeting acquired resistance by bacteria and with strategies to use antibiotics more effectively. Altogether, the lack of such an efficient approach to eradicate biofilms contributes to a relatively high incidence of treatment failure(24)

Our focus as part of providing alternative and new strategies for the prevention and treatment of medical device-associated infection is to devise local therapeutic delivery regimens based on a clinically relevant understanding of bacterial dynamics (growth and resistance acquisition) and with a realistic expectation of antibacterial activity. This understanding can help us devise implants tailored to deliver the types and amounts of drugs specific to the expected ‘bacterial state’. It can also guide us in combining local delivery with other antibacterial tools at our disposal for maximum treatment success.

Combination therapeutic strategies involving known antibiotics with other novel and repurposed non-antibiotic drugs are being explored to overcome the burden of bacterial resistance(30,31). We have been investigating the effect of common peri-surgical non-antibiotic therapeutics with antibiotics such as gentamicin which is one of the most used local antibiotics in orthopaedics due to its broad-spectrum activity(32). Our findings were that the non-steroidal anti-inflammatory ketorolac tromethamine is synergistic with gentamicin(33). This strategy incorporating drugs that are already in peri-surgical use is significant because it can potentially increase treatment success without additional risks to the patients. In this study, we are investigating the effect of gentamicin and ketorolac on planktonic bacteria as well as nascent and mature biofilms of laboratory and clinical strains of SA and SE in clinically relevant concentration ranges. The synergistic activity was characterized by determining the improved MBEC for gentamicin in the presence of ketorolac and the effect of synergy on the biofilm morphology and physiology. Our results suggest strongly that clinically relevant therapeutics used for the same local indication should be included in models for more accurate antibacterial assessment.

## MATERIALS AND METHODS

### 1. Bacteria culture and maintenance

Control and clinical strains of Staphylococcus aureus (ATCC 12600, L1101) and Staphylococcus epidermidis (ATCC 35984, L1116) with varying antibiotic susceptibility profiles were used in this study (Additional file 1). All bacterial strains were thawed out from glycerol stocks stored at −80°C and were cultured in sterile tryptic soy broth (TSB) or on tryptic soy agar plates (TSA)for a period of 18–24 hours at 35°C to achieve optimum growth prior to all experiments. The concentration of overnight bacterial suspension was spectrophotometrically determined at 600nm and was enumerated using growth curves generated for each strain used in this study.

### 2. Susceptibility testing of *Staphylococcal strains.*

The minimum inhibitory concentration [MIC] for GS and KT was determined to evaluate the susceptible and resistant nature of the Staphylococcal strains according to the Clinical and Laboratory Standards Institute (CLSI) protocol M07-A10 as described here. Briefly, the micro broth dilution assay was set up in a 96-well plate. GS and KT drug stocks were serially diluted in the wells using sterile Mueller Hinton Broth media (MHB) and 100uL of diluted bacterial suspension (~10^5^ CFU) was added to each well containing a range of drug concentrations. Wells containing only bacteria and only blank media served as internal controls for the assay. The well plate was statically incubated at 35°C for a period of 18–24 hours. The minimum concentration which showed no turbidity was determined as the MIC of the drug.

The cumulative antibacterial effect of GS and KT on the Staphylococcal strains was determined by performing a checkerboard assay to measure the fractional inhibitory concentration as described previously(34). Briefly, the overnight bacterial cultures were diluted to ~10^5^ CFU and exposed to different ratios of GS and KT combinations with MIC of each drug being the highest concentration combination tested. Following overnight incubation at 35°C, the turbidity was visualized, and FIC indices were determined. The drug combination was considered synergistic if the ΣFIC<1.0(35).

### 3. Evaluation of in-vitro biofilm dynamics

Staphylococcal bacterial suspension [10^5^ CFU/ml] in 1 mL of Luria-Bertani (LB) broth was inoculated on 316 Stainless Steel (SS) plates [10 × 3 × 1mm] placed within 24-well plates. The SS plates were statically incubated for a period of 48 hours at 35°C. At each time point (6hrs, 24hrs, and 48hrs), the spent media was removed, and the SS plates were washed thrice using sterile 1× phosphate-buffered saline (PBS). The SS plates were transferred to 1.5 mL tubes and subjected to sonication for 40 minutes in 1 mL PBS to dislodge the adherent bacteria, The sonicate was then plated on tryptic soy agar plates and incubated for 18–24 hours at 35°C. The adherent bacteria count was determined the following day by the colony counting method.

### 4. Determination of Minimum Biofilm eradication concentration (MBEC)

Staphylococcal biofilms were grown for a period of 6hrs and 24hrs on 316 SS coupons as previously described (section 4). The spent media was removed at each timepoint respectively and the SS plates were washed thrice with PBS to remove all non-adherent bacteria. The SS plates were then placed in a fresh 24-well plate containing GS and/or KT drug concentrations prepared in 10% LB-supplemented PBS solutions. The GS concentration range tested was (1–500 g/mL) to determine the evolution of drug resistance in nascent (6hrs) and established (24hrs) biofilms The KT concentrations tested were fixed at 0.5mg/ml or 1mg/ml (6h biofilms) and 1mg/ml or 3mg/ml (24hr biofilms). Further to drug exposure for 24 hours at 35°C, the SS plates were gently rinsed thrice using PBS and were transferred to 1.5ml tubes containing 1ml PBS. The plates underwent sonication for 40 minutes and the adherent bacteria count was determined using the spread plate method. MBEC was determined as >3log_10_ reduction in adherent bacteria count.

The MBEC of the GS-KT combination was visualized using the BacLight^™^ Live/Dead assay (Invitrogen, USA). Briefly, drug-treated nascent and established biofilms on SS plates (two per drug condition) were stained with SYTO 9/propidium iodide dye mixture according to the manufacturer’s protocol (1.5uL of each dye per 1mL of sterile deionized water). 200uL of the diluted dye was dispensed on each of the stainless-steel plates for thirty minutes in the dark, followed by two washes with deionized water. The stained biofilms were viewed using fluorescent microscopy (Nikon Ti2 Eclipse, Nikon Instruments Inc, USA, 400x magnification). Each plate was inverted onto an oil droplet on the surface of a thin glass coverslip which was held in place by a coverslip holder (Electron Microscopy Sciences). Ten fields were imaged per condition, with five randomly selected fields per plate. Propidium iodide-stained cells and SYTO 9-stained cells were visualized using Texas Red and GFP filters respectively. The resulting TIFF images were analyzed and quantified using the Biofilm Viability Checker plugin for Fiji (ImageJ^™^) image analysis software(36), which determined the proportion of live and dead bacteria.

### 5. Molecular characterization of antibacterial activity of GS-KT

#### 5.1 Scanning electron microscopy

Scanning electron microscopy was performed on the adherent bacteria on SS plates using the protocol described previously(37). Briefly, the adherent bacteria were fixed using 2.5% glutaraldehyde in 0.1M PBS for 48 hours. The plates were then washed twice for 10 mins with PBS. The adherent bacteria were then treated with osmium tetroxide (OsO^4^) 2% + Ruthenium red 0.2% 1:1 solution for a period of 1hr. The samples were washed twice thoroughly with distilled water for 10 mins. Further to this the samples were treated with 1% Tannic acid for 30 mins and then washed twice with distilled water for 10 mins each. The prepared samples were imaged at 15–20 kV, high vacuum (Zeiss FESEM Ultra Plus).

#### 5.2 Gene expression analysis

Adherent bacteria from 6hrs and 24hrs biofilms were exposed to indicated concentrations of GS and/or KT. The bacteria were harvested from SS plates (n=10) for each condition. The sonicates were pooled and pelleted by centrifuging at 10,000 ×g for 10 mins. The pellet was then subjected to mechanical and enzymatic lysis and the total RNA was extracted using RNesay power biofilm kit protocol (Qiagen, Germany) for gram-positive bacteria. The RNA yield and quality were spectrophotometrically assessed using NanoDrop (Thermo Scientific, USA). RNA samples were converted to cDNA according to the iScript cDNA conversion kit protocol (Bio-Rad, USA). Real-time quantitative PCR was performed for *icaA*, *icaD*, *ebpS*, *vraR* genes for *S. aureus* and *icaA*, *icaD*, *atlE*, *vraR* for *S. epidermidis* using specific primers listed (Additional file 1). The Cq values were normalized to *S. aureus* and *S. epidermidis 16srRNA* expression respectively. Comparative gene expression analysis was performed using the 2^^(−ΔΔCt)^ method(38). No drug-treated 6hrs and 24hrs adherent bacterial gene expression served as control.

#### 5.3 Membrane fluidity analysis

The membrane fluidity was investigated using the Laurdan assay(39). Briefly, bacterial suspension [10^8^CFU/ml] was centrifuged at 6000 × g, 5 min and the pellets were washed thrice with sterile PBS. The bacterial pellet was resuspended in 10 M Laurdan reagent and incubated at dark for 10 mins. The stained bacteria were pelleted and washed multiple times to remove excess stains. 100 L of Laurdan-stained bacteria was then treated with indicated concentrations of GS and/or KT and the bound Laurdan fluorescence was read every 10 mins for 40 mins using a microplate reader. 50mM benzyl alcohol (xx) treated bacteria served as the positive control. The fluorescence was read at excitation 350nm and at two emission wavelengths 435nm and 500nm. The laurdan generalized polarization (GP) was calculated using the following equation(40).


$$Laurdan\;GP=\frac{i435nm\;-\;i500nm}{i435nm\;+\;i500nm}$$

### 6. Statistical analysis

The gene expression studies were performed in triplicates and the non-parametric dataset was statistically analyzed using a two-sided Wilcoxon Rank Sum Test. The p-value was calculated and the lowest significant score of 0.1 was considered statistically significant.

## RESULTS

### Gentamicin-ketorolac combination demonstrated synergistic antibacterial action against planktonic Staphylococci.

To determine the cumulative antibacterial effect of GS and KT, we performed the checkerboard assay to evaluate fractional inhibitory concentration (FIC). Minimum inhibitory concentration (MIC) measurements for GS and KT were performed on the laboratory and clinical staphylococcal strains as a prerequisite to FIC testing. The MIC values validated the gentamicin sensitivity level of all the strains (Table 1). The control SA demonstrated the lowest MIC for GS compared to that of the control SE and the clinical strains. KT demonstrated low growth inhibitory activity against the clinical strains compared to that against reference strains. The checkerboard assay was designed for each strain using the MIC values of the single drugs. GS-KT combination demonstrated ΣFIC<1 for all staphylococcal strains indicating a synergistic/additive antimicrobial effect against planktonic bacteria which confirmed our previous findings for an expanded set of organisms and strains (Table 2)(33).

### Increasing biofilm maturity confers increased resistance to gentamicin for susceptible and resistant strains.

**Fig. 1.**
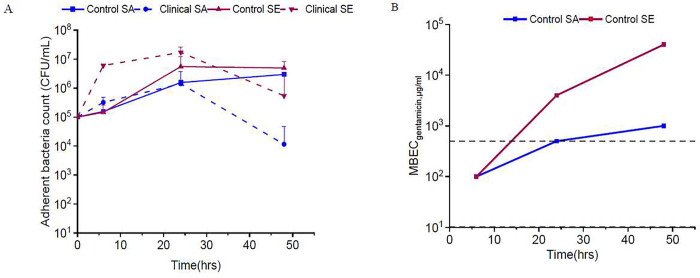
Staphylococcal biofilm growth dynamics and gentamicin resistance evolution over time. **(A)** Adherent bacteria count was determined to evaluate the biofilm dynamics of control (ATCC 12600, ATCC 35984) and clinical (L1101, L1116) Staphylococcal strains. **(B)** MBEC gentamicin evolution was determined over a period of 48hrs for both control and clinical strains. The maximum concentration of gentamicin tested was capped at 500 g/ml. Both 6hr and 24hr biofilms of clinical strains demonstrated MBEC>500 μg/mL.

To characterize the biofilm dynamics and the role of biofilm in imparting resistance to gentamicin, in-vitro biofilm models were established on 316L stainless steel plates. The differences in growth dynamics among staphylococcal species were characterized for both reference and clinical strains. The control SA strain demonstrated a steady increase in adherent bacteria counts over 48 hours. In contrast, the clinical SA strain showed a decline in viable (adherent) bacteria count after 24 hours. The control SE strain, which possesses strong biofilm-forming capability, showed a sharp increase in viable adherent bacteria under 24 hours and maintained high adherent viable bacteria counts even after 48 hours. On the contrary, the clinical SE strain showed a drastic increase in adherent bacteria count in just under 6 hours and a gradual decrease was observed after 24 hours (Figure 1A).

Biofilms show decreased susceptibility to antibiotics reducing the efficacy of treatments(41). The role of biofilm dynamics in the significant reduction of susceptibility has not been well characterized with respect to medical device-associated infections. The development of resistance to GS treatment for in-vitro-grown biofilms was determined by exposing the biofilms to a range of GS concentrations. The MBEC_gentamicin_ (>3 log reduction) for a 6hr-grown biofilm of the reference SA strain was 100 ± 20 μg/ml, which subsequently increased to >500 μg/mL at 48h of growth. This result showed that increased biofilm maturity conferred increased drug resistance to an otherwise susceptible strain. The biofilm of the reference SE strain grown for 6hr demonstrated an MBEC of 100 ± 20 μg/ml, which drastically increased to >500 g/ml for 24hr and 48hr biofilms. On the other hand, biofilms of the clinical staphylococcal strains grown for 6, 24, and 48hrs all exhibited MBEC>500 μg/mL. The data showed the compounded effect of inherent and acquired resistance for these strains (Figure 1B). Based on these data, we designated 500 μg/ml of gentamicin as a clinically feasible maximum concentration of interest. For the following experiments evaluating synergy, we designated biofilms grown for 6 hrs as ‘nascent’ and those grown for 24 hours as ‘established’ based on the evolution of resistance against gentamicin.

### Ketorolac enhances the activity of gentamicin against acquired resistance.

**Fig. 2.**
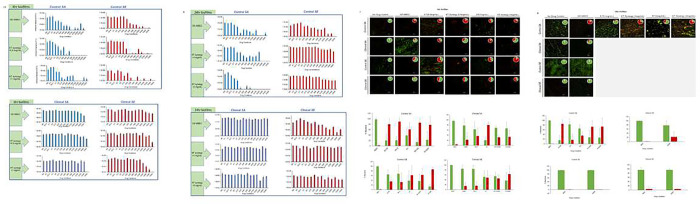
Addition of ketorolac enhances the activity of gentamicin against biofilm maturity-associated resistance in staphylococcal biofilms **(A)**Effect of KT synergy (0.5mg/mL and 1mg/mL) on nascent control and clinical staphylococcal biofilms. **(B)** Effect of KT synergy (1mg/mL and 3mg/mL) on established control and clinical staphylococcal biofilms. **(C)** Effect of KT synergy on nascent and **(D)** established biofilms observed using Live/Dead staining (scale bar= 50μM). The live/dead adherent bacteria percentage was quantified and represented as a bar plot. The error bars represent the standard deviation (n=10).

We investigated the effect of the use of KT to determine the range of concentrations for gentamicin that are efficacious against the strains with different susceptibility including those with increased inherent or acquired resistance. We used 0.5 and 1 mg/ml KT for nascent (6hr) biofilms and 1 and 3 mg/ml KT for established (24hr) biofilms. All of these concentrations were below the MIC of KT (Table 1) and in the clinically feasible range for pain management by KT administration(42,43). The addition of 0.5 and 1 mg/ml of ketorolac significantly decreased the MBEC for the nascent biofilms of all tested strains (Figure 2A). For the bacteria in the established biofilms of the control SA strain with increased acquired gentamicin resistance, there was a >3log reduction with the addition of 1 and 3 mg/mL KT at gentamicin concentrations of 40 and 20 μg/mL, respectively. The bacteria in the biofilms of the inherently resistant clinical strains were not eradicated at gentamicin concentrations below the threshold concentration with the addition of indicated KT (Figure 2B). The MBEC values for gentamicin against the nascent and established biofilms with and without the addition of KT are summarized in Table 3 and were used for all subsequent experiments evaluating the extent and mechanism of the synergy.

The synergistic antibiofilm effect was confirmed in real-time by quantifying the viability of the biofilm using the Live/Dead assay. The *nascent* biofilms of the control SA, SE, and clinical SE strains demonstrated viability loss with the addition of KT, which was either comparable or more than the observed viability loss for biofilms exposed to GS treatment alone at MBEC. For the inherently resistant clinical SA strain, both GS and synergistic GS-KT combination treatment did not show a real-time impact on bacterial viability. The treatment of the clinical SA biofilm with 1 mg/mL of KT alone without GS demonstrated viability loss (Figure 2C), suggesting that the variability of the bacterial eradication capability of KT needs further study for multiple strains including multi-drug resistant ones. The synergistic action against biofilm-associated acquired resistance was also determined for *established* biofilms of the reference SA strain. The addition of 1 and 3 mg/mL KT in combination with 40 μg/mL and 20 μg/mL of GS, respectively, reduced bacterial viability which was comparable to the biofilms treated with GS at MBEC (100μg/mL) (Figure 2D), in line with the plate culture. On the other hand, the biofilms of the inherently resistant strains (clinical SA, control SE, and clinical SE) showed no significant biofilm viability loss in the presence of GS.

### The addition of KT interferes with extracellular matrix formation and biofilm morphology.

**Fig. 3.**
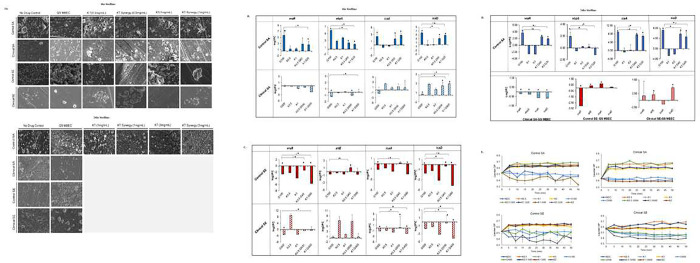
Characterization of GS-KT synergistic antibiofilm activity against staphylococcal biofilms. **(A)** Scanning electron micrographs of nascent and established biofilms exposed to GS MBEC and KT synergy combinations (scale bar=2μM, magnification= 2.5KX). Gene expression analysis of *vraR*, *icaA*, *icaD*, *ebpS*, and *atlE* genes in nascent [**(B), (C)],** and established biofilms **[(D)]** of control and clinical staphylococcus. Error bars represent the standard deviation of 3 replicates. (Wilcoxon rank sum test; *indicates p-value of 0.1). **(E)** Membrane fluidity (Laurdan GP) was determined for the control and clinical staphylococcal strains exposed to GS MBEC and KT synergy combinations. The line graphs denote laurdan GP values plotted over a period of 40 minutes. Error bars represent the standard deviation (n=3)

Scanning electron microscopy was performed to visually examine nascent and established biofilms when exposed to GS and GS together with KT. The images were qualitatively analyzed for the differences in the extracellular matrix density and morphology, bacterial aggregation pattern, density, and the integrity of the cell wall. A series of images were taken after exposing the biofilms grown on SS plates to the effective concentration of GS, synergistic combination of GS/KT and control concentration of KT indicated in Table 3. The untreated biofilms exhibited more intact bacterial aggregates attached to the SS plate. The connective ECM structures were more abundant in the biofilms of the control strains compared to those of the clinical strains (Figure 3A).

*Nascent biofilms of control SA* treated with K1 synergy concentration showed sparse bacterial density and an overall reduction of bacteria and bacterial aggregation. These observations were similar to those in the biofilms exposed to GS only at MBEC. Morphologically, the K1 synergy combination resulted in more fractured extracellular matrix (ECM) structures compared to other conditions. Additionally, the bacterial spheres attached to the ECM structures were reduced in size compared to the bacteria seen in the GS MBEC-treated and no drug-treated conditions.

For the *nascent biofilms of clinical SA*, the K1 synergy condition showed a small deposition of ECM with little to no attached cells and the remnants of deflated spheres of individual bacteria. These features were similar to those of the sparsely distributed bacteria exposed to GS only at MBEC (Figure 3A). Thus, the imaging confirmed that exposing nascent biofilms to synergistic combinations of GS/KT (where GS concentration<MBEC) resulted in similar biofilm features to when they were exposed to GS only at MBEC.

In *nascent biofilms of control SE*, there was little ECM production (Figure 3A). Control SE biofilm exposed to K1 synergy concentration demonstrated sporadic ECM structures studded with a few viable bacteria. This was in stark contrast to the biofilms exposed to GS only at MBEC, which showed dense ECM with copious embedded bacteria and bacteria agglomerates.

*The nascent biofilms of the clinical SE strain* exposed to the K1 synergy concentration exhibited sparsely distributed matrix debris and deflated bacteria (presumably non-viable). These features were similar to those of biofilms exposed to GS only at MBEC (Figure 3A).

*The established biofilms of control SA* showed greater surface coverage of the SS plate compared to that of the nascent biofilms, with intact bacterial populations and connected ECM structures (Figure 3B). Although biofilms treated with GS only at MBEC appeared thinly dispersed with reduced viability, the biofilms exposed to the K3 synergy concentration showed larger areas of fragmented ECM structures in addition to the reduced viability of bacteria.

*The established biofilms of clinical SA* showed deflated, dispersed bacteria with little ECM when exposed to GS only at MBEC. *The established biofilm of the control SE* showed denser bacterial agglomerates with little ECM and *the established biofilm of the clinical SE* showed similar features to that of the clinical SA. There were no synergistic combinations of GS/KT for GS concentrations less than 500 μg/ml for established biofilms of these three strains. The most visible effect of the presence of KT was the increased production of ECM. The synergistic use of GS/KT affected the bacterial viability and decreased the structural integrity of the ECM for the biofilms susceptible to it. These results also suggested an influence of ketorolac on the balance between resistance acquisition and other processes such as matrix production.

### GS-KT synergy subverts bacterial stress response and alters biofilm properties.

To elucidate the changes in bacterial stress responses triggered by exposure to GS at MBEC and at synergistic combinations with KT, gene expression studies were performed (Figures 3C and 3D). The effect of exposing the bacteria to MBEC on the expression of biofilm-associated *icaA, icaD* genes, bacterial adhesion-associated *ebpS, and atlE* genes (for *S. aureus* and *S. epidermidis,* respectively) and peptidoglycan biosynthesis-associated *vraR* gene were studied.

*In nascent biofilms of control SA*, exposure to GS at MBEC significantly increased the *vraR*, *ebpS*, *icaA,* and *icaD* expression when compared to that with no drug exposure. Concurrently, exposure to the K0.5 synergy and K1 synergy concentrations also demonstrated upregulation for all the genes to a lesser degree when compared to the expression of biofilms exposed to GS at MBEC. The data indicated similarities in the stress responses triggered by GS at MBEC and the synergistic concentrations with KT, which agreed with the qualitative visual cues obtained using SEM such as the size and density of bacterial aggregates. The presence of KT alone did not show a difference in gene expression except for that of eb*pS*, which revealed that adhesion was upregulated (>1.5 fold) for all conditions.

*In nascent biofilms of clinical SA*, the expression of the four studied genes was not changed when the bacteria were exposed to GS at MBEC, supporting the response expected from this resistant strain in the presence of gentamicin. However, in the presence of KT, and its synergistic combinations with GS, *icaD* expression was significantly upregulated (~3-fold) as well as *vraR* (>2-fold), and *icaA* (1-fold) and no change was observed for *ebpS*. This data supported the real-time viability loss observed in the presence of KT for nascent clinical SA biofilms at high GS concentrations (Figure 2C). The increase of icaA and icaD gene expression supported the SEM observations for the clinical SA, which showed more aggregation of bacteria. The size of the bacteria was small, which may be attributed to increased drug resistance and higher vraR expression(44).

*In nascent biofilms of control SE*, bacteria with exposure to GS at MBEC demonstrated significant downregulation of *vraR* (>2-fold), *icaA* (1-fold), and *icaD* (>2-fold) genes. The exposure to the K1 synergy combination (KT 1mg/mL +GS 60 g/mL) triggered a further reduction in gene expression levels for *vraR* (>5-fold), *icaA* (2-fold), and *icaD* (4-fold), revealing a notable enhancement of antibiofilm properties of GS with the addition of KT (Figure 3C). The gene expression data correlated with observations from SEM images and viability data exhibiting significantly diminished colony size, less bacterial aggregates, reduced slime production, and viability loss respectively for nascent control SE biofilms (Figure 2C and 3A). On the contrary, adhesion-associated *atlE* showed a slight but significant increase when compared to GS at MBEC and no drug control. The overall increased expression of *atlE*, which is the most prominent SE adhesin, could be attributed to the increased adhesion capability of SE strains(45).

*Nascent biofilms of clinical SE* exhibited a significant reduction in *icaA, icaD,* and *vraR* in the presence of GS at MBEC compared to those without drug exposure. The presence of KT in the synergistic combinations, specifically K1 synergy, increased the deregulation of *icaA* and *icaD* genes compared to biofilms exposed to GS at MBEC. The synergistic combinations did not show a difference in the regulation of the *vraR* gene expression compared to that of the biofilms exposed to GS at MBEC. This indicated that the addition of KT suppressed the matrix production triggered by GS. The expression of the adhesion-related atlE gene was upregulated by exposure to the K0.5 synergy combination compared to exposure to GS at MBEC; however, the change was not statistically significant (Figure 3C). The data agreed with the visual observations of pronounced antibiofilm activity exerted by the synergistic combination of GS and KT (Figures 2C and 3A).

For the *established biofilms of the control SA strain*, exposure to GS at MBEC and to K1 and K3 synergy combinations significantly upregulated the expression of *vraR* (>2-fold), *icaA* (8-fold), and *icaD* (>5-fold) when compared to those without drug exposure (Figure 3D). The bacterial molecular responses triggered by the exposure to both GS at MBEC and its synergistic combinations with KT, as determined by gene expression profiles, were consistently similar in established biofilms. This behavior was markedly different from that of nascent biofilms, where the addition of KT significantly reduced the gene expression levels of *ebpS* compared to biofilms exposed to GS at MBEC, which indicated compromised adhesion in biofilms exposed to KT synergy combinations. The data obtained aligned with the viability assay and SEM observations indicating GS and KT synergistically work to impact biofilm adhesion, matrix formation, and biofilm-associated resistance to antibiotics (Figures 2D and 3B).

Due to the absence of effective synergistic combinations against the established biofilms of *clinical SA, control SE, and clinical SE strains,* the gene expression analysis was performed only for biofilms exposed to GS at MBEC. For the clinical SA, there were low levels of expression for all four genes but there were significant differences when compared to biofilms with no drug exposure. In contrast, *vraR* expression in *control SE biofilms* was significantly downregulated (>2.5-fold) in the presence of GS at MBEC along with a <1-fold increase for *atlE* and *icaA* genes. In the *clinical SE strain,* GS exposure at MBEC triggered a significant but small upregulation effect for atlE (0.5-fold) and icaD (1-fold) expression (Figure 3D). The data validated the observations from the live/dead viability assay and SEM indicating an overall subdued response of these strains against GS.

### The addition of KT triggers staphylococcal membrane rigidification.

The antibacterial investigation of exposure to KT in addition to GS and the demonstration of efficacy, especially in hindering acquired resistance of nascent biofilms, suggests an alternative mechanism of action for KT. Antibiotics are known to impact bacterial membrane fluidity during their course of action(46,47). The antibacterial activity greatly depends upon the capability of antibiotics to insert themselves into the bacterial membrane or access the inner physiological system through specific channels across the membrane(48,49). To understand the mechanism of action of GS at MBEC and its synergistic combinations of KT on the bacterial membranes of staphylococcal strains, bacterial membrane fluidity was determined using Laurdan fluorescence(39). The addition of KT dramatically increased the Laurdan GP values (>0.5) exhibiting membrane rigidifying properties regardless of the presence of GS. Interestingly, the GS at MBEC-exposed bacteria showed average Laurdan GP values close to <0.5 indicating little to no fluidizing properties of GS within the time frame of the experiment (40 minutes) when compared to no-drug control. Bacteria exposed to benzyl alcohol as a membrane fluidizer served as the positive control (Laurdan GP values <0.4) (Figure 3E). The data validated the nature of killing kinetics of gentamicin which is known to be partially concentration-dependent and that higher concentrations of gentamicin do not aid in additional eradication due to adaptive resistance mechanisms(50,51). A novel membrane-rigidifying molecular action of KT was revealed which seems to facilitate the synergistic antibacterial action with GS.

## Discussion

The elucidation of the translational value of antibiotic-analgesic synergistic use in treating infections holds significant promise for improving patient and overall healthcare outcomes. Local administration of antibiotics is used in clinical practice to prevent infections(52) and of local anesthetics/analgesics to address peri-surgical pain(53–55). Previously we showed that several commonly used analgesics and NSAIDs yield pronounced synergistic/additive antibacterial effects against PJI-causing planktonic *Staphylococci* when used in combination with antibiotics *in vitro*(33). The outcomes of PJI are often complicated by bacterial strains harboring inherent antibiotic resistance and biofilm-forming capabilities which significantly impact the antibacterial activity of drugs(56). The combined use of antibiotics and analgesics to target infections would aid in reducing the inappropriate use of antibiotics and hindering the development of antibiotic resistance. In this study, we are exploring and optimizing the application of the NSAID ketorolac in combination with the antibiotic gentamicin, to synergistically enhance its efficacy, allowing for better control of staphylococcal biofilms.

Synergy has been investigated and reported largely for multiple antibiotics(57). In the face of the emergence of multi-drug resistant organisms, antibiotic synergy with non-antibiotics has also gained interest(31,58) Most of the work has focused on the synergy between antibiotics and non-conventional antimicrobial strategies such as the use of bacteriophages, antimicrobial peptides, and small molecules, inhibiting vital bacterial processes(59–62). Recently, there has also been considerable interest in repurposing non-antibiotic drugs for the purpose of infection prevention and management(63,64). In a post-operative care setting, local application of antibiotics and non-antibiotic drugs are common to prevent the onset of PJI and pain management. However, little is known about the repurposing of nonantibiotic drugs (locally) to enhance the activity of existing antibiotics and no combinations have been investigated specifically for indications related to periprosthetic (bone) infections.

One of the major challenges encountered is the identification and the physiological profiles of the bacterial strains causing PJI. The complexity of infections is determined by virulence, biofilm formation, innate antibiotic resistance, and persistence. Timely diagnosis is paramount in understanding the risk and severity of the infection at hand and in designing the best possible therapeutic approaches that would result in the effective eradication of the infection without the development of further resistance or persister populations. Current approaches focus on delivering a pre-determined cocktail of antibiotics for PJI patients without clarity about the status of infecting organisms(58,65). This ‘one size fits all’ therapeutic strategy has not hindered the increasing incidence in infections leading to significant morbidity and mortality(66,67). Even though antibiotic treatment remains the gold standard to combat infections, the presence of inherent resistance and the development of acquired resistance due to biofilm formation are problems that warrant alternative solutions. The use of non-antibiotic drugs in conjunction with antibiotics to improve overall antibiotic activity can be an effective approach to enhancing the activity of currently used antibiotics without increasing the risk of overall antibiotic resistance. Here we explored the combined antibacterial activity of two drugs used in local applications for total joint arthroplasty to explore the enhancement of antibacterial activity against nascent (6hr) and mature (24hr) staphylococcal biofilms. Different strains harboring varying resistance profiles were used to understand the range of applicability of the proposed dual use of gentamicin and ketorolac against eradicating biofilms of varying risk and severity.

We used a ‘low risk’ gentamicin and vancomycin-susceptible staphylococcal strain and a ‘high risk’ gentamicin-resistant and vancomycin susceptible/intermediate staphylococcal strain to simulate a range of infection severity *in vitro*. The infection was characterized by determining the growth dynamics over 48 hours which significantly differed among the tested strains. There were strain-specific differences in the biofilm formation rate and viable bacterial recovery at each time point for in-vitro-grown biofilms. When the nascent and established biofilms were subjected to a range of gentamicin concentrations, an increase in gentamicin resistance commensurate with increasing biofilm maturity was observed. Thus, using two strains with differing inherent resistance in combination with their differing time-dependent acquisition of resistance due to biofilm formation gave us a wide range of ‘bacterial states’ against which to test synergy.

The primary goal of this study was to determine whether a non-antibiotic compound can be used to work in tandem with the antibiotic to overcome biofilm-associated acquired resistance. To this end, low concentrations (0.5, 1, and 3 mg/mL) of ketorolac close to clinical dosing guidelines were used to determine the synergistic action. The MBEC was determined, and live/dead staining and scanning electron microscopy were utilized to visualize the phenotypical traits of bacteria exposed to synergistic concentrations of ketorolac with gentamicin. The addition of ketorolac significantly reduced the GS at MBEC required to eradicate nascent and established ‘low risk’ biofilms and affect the viability for nascent ‘high risk’ biofilms. Interestingly, the data also showed considerable differences between the antibiofilm effect exerted by synergistic combinations on *S. aureus* and *S. epidermidis*. The pronounced antibiofilm effect of the gentamicin-ketorolac combinations significantly altered the biofilm morphology and physiology in all the exposed biofilms. The findings from this study reveal that the synergistic use of ketorolac with gentamicin, for example, that is eluted from antibiotic-eluting bone cement could be evaluated as a prophylactic measure to reduce the antibiotic load and increase treatment efficacy.

The critical factors for *staphylococcal* biofilm formation are the presence of abiotic surfaces and damaged peri-implant tissues *in vivo.* The bacterial colonization process is associated with the expression of several adhesins, slime production, and stress-induced pathways that aid the bacteria to effectively attach to surfaces, evade host response to infection, and resist antibiotic treatments. Understanding the regulation of these molecular events, specifically in response to the presence of antibacterial agents, is crucial to determining the antimicrobial dynamics. Gene expression studies were performed to understand the molecular behavior of staphylococci in the presence of synergistic combinations of gentamicin and ketorolac. *S. aureus* has been shown to induce cell wall-associated gene expression in the presence of antimicrobial agents(68–70). Moreover, the cell wall responses are directly linked to the emergence of small colony variants and antibiotic resistance(44,71,72). In our study, we observed that the bacterial response to restore the cell wall (vraR expression) was overall subdued in the presence of GS-KT, which was corroborated by the diminished cell size observed under SEM. The absence of such a crucial bacterial response in the presence of GS-KT to maintain the cell wall components could prove advantageous for gentamicin to gain easy access into bacterial cells due to poor cell wall integrity. Besides cell wall-associated expression changes, adhesion and biofilm formation are vital processes triggered during pathogenesis. *EbpS* and *atlE are* well-established adhesin markers for SA and SE biofilms that aid in direct interaction with host factors and surfaces in the initial bacterial interactions to promote attachment(73–75). In our study, GS treatment markedly increased adhesin expression in control SA nascent biofilm but was reduced with the addition of KT. This data strongly implies that the presence of commonly used gentamicin could potentially increase the bacterial attachment in a ‘low risk’ infection while KT addition can reduce this effect. However, in clinical SA biofilms, the expression was not significantly altered correlating with its expression being tied to the presence of soluble elastins(74). In SE biofilms irrespective of maturity, adhesin expression did not show an increased expression which correlated with it being important later in the biofilm cycle(45). Biofilm formation, which is mediated by the icaADBC locus(76), was found to be differentially regulated in a strain-dependent manner by GS-KT synergy. Among the two strains, *SE* is known to be the high slime producer clinically(77,78)and it was interesting to observe the drastic deregulation of icaA and icaD gene expression triggered by GS and GS-KT combinations. This data strongly emphasizes the potential of the synergistic application against biofilm formation as this is the major virulence factor for pathogenesis(77). In contrast, exposure to GS at MBEC and GS-KT showed increased expression of icaA and icaD in nascent and mature SA biofilms.

This data aligned with other studies reporting increased staphylococcal biomass in the presence of gentamicin(79). The gene expression overall complemented the SEM observations to a certain extent, but additional studies on other genes mediated by the ica locus are needed to reveal if the synthesized slime translocation and deacetylation pathways are affected by drug treatment(80). The nutrient-dependent uptake and activity of gentamicin in the presence of ketorolac should also be evaluated to further understand the synergy dynamics. These preliminary findings aid in the understanding of the mechanism of action of GS-KT synergy. For all the ‘high-risk’ mature biofilms, where the GS-KT combinations were ineffective, alternative analgesic and anti-inflammatory combinations with known antibiotics are being explored.

The gene expression data regarding the cell wall biosynthesis response of bacteria in the presence of GS at MBEC and GS-KT revealed that the addition of ketorolac significantly subdues or negatively regulates the bacterial pathways to restore membrane integrity. The bacterial lipid bilayers consist of rigid and fluid domains that determine the bacterial membrane fluidity, and they adapt quickly to changing environments(81). Laurdan reagent is capable of intercalating to the bacterial lipid bilayer and can assess the membrane fluidity based on the number of water molecules around the laurdan molecule(39,82). This attribute makes it an excellent probe to understand the mechanism of antimicrobial action. Aminoglycosides are internalized due to the presence of proton motive force (PMF) and the influx of gentamicin is known to cause increased membrane fluidity(83). Biofilm formation decreases the overall metabolic activity and growth leading to a diminished PMF imparting resistance to these antibiotics(84). Biofilm maturity is a factor in increased resistance to antibiotics and it is highly likely due to a diminished PMF(85). Recent reports have revealed that non-antibiotic agents can induce PMF-independent uptake of aminoglycosides by altering membrane potential(83). In our study, the addition of KT was primarily found to rigidify bacterial membranes in a concentration-independent manner. The data was similar to that of the antibiotic daptomycin which decreases membrane potential (dose-dependently) by clustering lipid domains due to its insertion into the bacterial membrane(86,87). These observations strongly suggest that the membrane destabilization caused by KT could improve the uptake of gentamicin into the biofilms in a PMF-independent manner and thereby target the biofilm maturity associated-gentamicin resistance.

Our results firstly showed possible differences in PJI biofilms *in vitro* and the possible application of stratifying the risk of infections while modeling clinically relevant situations. The adaptation of such a risk stratification strategy can enable the design of drug delivery vehicles and profiles according to the inherent resistance profile, biofilm composition, and molecular status of bacteria to improve treatment outcomes. Our study also revealed the potential of the synergistic use of an analgesic in combination with an antibiotic as a novel prophylactic approach to prevent PJI. The design of functional implants for the local sustained delivery of antibiotics as well as the delivery of synergistic combinations of drugs can be possible based on our work on polymeric drug delivery devices(88–91).

Our study is limited to in-vitro culture: there is very little known about the progression of in-vivo infections, especially in the early stages where prevention and treatment may be more probable. The diagnosis of infections *in vivo* is characterized by clinical symptoms and there are gaps in our knowledge about the characterization of the bacterial state and its changes over time. Thus, further study to understand the relationship between bacterial populations grown *in vivo* and *in vitro* is required. Similarly, the efficacy of antibiotic drugs can be affected by other factors in the in-vivo environment such as the binding of proteins and the clearance rate from the local environment. Finally, the host immune system has an integral role in determining the efficacy of any administered antibiotics as the major force in detecting, disarming, and clearing infections, which were not modeled in our study.

## Conclusion

In summary, the synergistic effect of post-operative anti-inflammatory drugs enhancing the antibacterial activity of common prophylactic antibiotic gentamicin was shown for the first time. Our findings also emphasize the importance of infection risk assessment to be used as a tool to design better prophylactic and therapeutic approaches.

## Figures and Tables

**Table 1:** MIC values for all the strains.

Bacteria		Strains	MIC gentamicin (μg/mL)	MIC ketorolac (mg/mL)
*Staphylococcus aureus*	Control SA	ATCC 12600	1	8±4
	Clinical SA	L1101	>16	128±2
*Staphylococcus epidermidis*	Control SE	ATCC 35984	>16	8±4
	Clinical SE	L1116	>16	128±2

**Table 2:** FIC indices for all the strains

Bacteria	Strains	Gentamicin-Ketorolac [ΣFIC]
*Staphylococcus aureus*	ATCC 12600	0.8
	L1101	0.8
*Staphylococcus epidermidis*	ATCC 35984	0.9
	L1116	0.8

**Table 3:** GS MBEC values for nascent and established biofilms with and without the addition of indicated concentrations of KT

Biofilm maturity	Bacteria	Strain	MBEC (GS, μg/mL)	+0.5 mg/mL KT	+1mg/mL KT
Nascent (6hr)	*S. aureus*	ATCC 12600	100	60	20
		L1101	>500	500	500
	*S. epidermidis*	ATCC 35984	200	40	60
		L1116	>500	500	400
**Biofilm maturity**	**Bacteria**	**Strain**	**MBEC (GS, μg/mL)**	**+1mg/mL KT**	**+3mg/mL KT**
Established (24hr)	*S. aureus*	ATCC 12600	100	40	20
		L1101	>500	-	-
	*S. epidermidis*	ATCC 35984	400	-	-
		L1116	400	-	-

## Data Availability

The data supporting the conclusions of this article are included within the article. More information on materials and methods are provided in Additional File 1.

## References

[1] Kurtz SM, Lau E, Watson H, Schmier JK, Parvizi J. Economic burden of periprosthetic joint infection in the United States. J Arthroplasty. 2012 Sep;27(8 Suppl):61–5.e1. 22554729 10.1016/j.arth.2012.02.022

[2] Wimmer MD, Hischebeth GTR, Randau TM, Gathen M, Schildberg FA, Fröschen FS, Difficult-to-treat pathogens significantly reduce infection resolution in periprosthetic joint infections. Diagn Microbiol Infect Dis. 2020 Oct 1;98(2):115114. 32712505 10.1016/j.diagmicrobio.2020.115114

[3] Visperas A, Santana D, Klika AK, Higuera-Rueda CA, Piuzzi NS. Current treatments for biofilm-associated periprosthetic joint infection and new potential strategies. Journal of Orthopaedic Research. 2022 Jul 6;40(7):1477–91. 35437846 10.1002/jor.25345 PMC9322555

[4] ping Chen J, hsiang Chang C, chih Lin Y, hsun Lee S, nung Shih H, Chang Y. Two-stage exchange Arthroplasty for knee Periprosthetic joint infection exhibit high infection recurrence rate in patients with chronic viral hepatitis. BMC Musculoskelet Disord. 2021 Dec 12;22(1):538. 34118906 10.1186/s12891-021-04416-0 PMC8199816

[5] Egbulefu FJ, Yang J, Segreti JC, Sporer SM, Chen AF, Austin MS, Recurrent Failures After 2-Stage Exchanges are Secondary to New Organisms Not Previously Covered by Antibiotics. Arthroplast Today. 2022 Oct;17:186–191.e1. 36254212 10.1016/j.artd.2022.07.015 PMC9568676

[6] Sabah SA, Shearman AD, Alvand A. An update on prosthetic joint infection for UK trainees. Surgery (Oxford). 2021 Nov;39(11):748–51.

[7] Kurtz SM, Lau EC, Son MS, Chang ET, Zimmerli W, Parvizi J. Are We Winning or Losing the Battle With Periprosthetic Joint Infection: Trends in Periprosthetic Joint Infection and Mortality Risk for the Medicare Population. J Arthroplasty. 2018 Oct 1;33(10):3238–45. 29914821 10.1016/j.arth.2018.05.042

[8] Parvizi J, Tan TL, Goswami K, Higuera C, Della Valle C, Chen AF, The 2018 Definition of Periprosthetic Hip and Knee Infection: An Evidence-Based and Validated Criteria. J Arthroplasty. 2018 May;33(5):1309–1314.e2. 29551303 10.1016/j.arth.2018.02.078

[9] Lee J, Park H, Bae J, Hyun H, Kim S. Current Diagnostic Methods for Periprosthetic Joint Infection. Biomedical Science Letters. 2022 Mar 31;28(1):1–8.

[10] Castelli CC, Gotti V, Ferrari R. Two-stage treatment of infected total knee arthroplasty: two to thirteen year experience using an articulating preformed spacer. Int Orthop. 2014 Feb 26;38(2):405–12. 24464017 10.1007/s00264-013-2241-6 PMC3923954

[11] Tan TL, Goswami K, Fillingham YA, Shohat N, Rondon AJ, Parvizi J. Defining Treatment Success After 2-Stage Exchange Arthroplasty for Periprosthetic Joint Infection. J Arthroplasty. 2018 Nov;33(11):3541–6. 30100137 10.1016/j.arth.2018.06.015

[12] Rottier W, Seidelman J, Wouthuyzen-Bakker M. Antimicrobial treatment of patients with a periprosthetic joint infection: basic principles. Arthroplasty. 2023 Mar 2;5(1):10. 36864531 10.1186/s42836-023-00169-4 PMC9979546

[13] Kapadia BH, Berg RA, Daley JA, Fritz J, Bhave A, Mont MA. Periprosthetic joint infection. The Lancet. 2016 Jan 23;387(10016):386–94. 10.1016/S0140-6736(14)61798-0 26135702

[14] Lentino JR. Prosthetic Joint Infections: Bane of Orthopedists, Challenge for Infectious Disease Specialists. Clinical Infectious Diseases. 2003 May;36(9):1157–61. 12715311 10.1086/374554

[15] Yang J, Parvizi J, Hansen EN, Culvern CN, Segreti JC, Tan T, 2020 Mark Coventry Award: Microorganism-directed oral antibiotics reduce the rate of failure due to further infection after two-stage revision hip or knee arthroplasty for chronic infection: a multicentre randomized controlled trial at a minimum of two years. Bone Joint J. 2020 Jun;102-B(6_Supple_A):3–9. 10.1302/0301-620X.102B6.BJJ-2019-1596.R1 32475278

[16] Triffault-Fillit C, Ferry T, Laurent F, Pradat P, Dupieux C, Conrad A, Microbiologic epidemiology depending on time to occurrence of prosthetic joint infection: a prospective cohort study. Clinical Microbiology and Infection. 2019 Mar 1;25(3):353–8. 29803842 10.1016/j.cmi.2018.04.035

[17] Gristina AG, Naylor P, Myrvik Q. Infections from biomaterials and implants: a race for the surface. Med Prog Technol. 14(3–4):205–24. 2978593

[18] Chu L, Yang Y, Yang S, Fan Q, Yu Z, Hu XL, Preferential Colonization of Osteoblasts Over Co-cultured Bacteria on a Bifunctional Biomaterial Surface. Front Microbiol. 2018 Oct 2;9. 10.3389/fmicb.2018.02219 PMC6176048 30333796

[19] Shiels S, Mangum L, Wenke J. Revisiting the “race for the surface” in a pre-clinical model of implant infection. Eur Cell Mater. 2020 Jan 29;39:77–95. 31995226 10.22203/eCM.v039a05

[20] Coenye T, Bové M, Bjarnsholt T. Biofilm antimicrobial susceptibility through an experimental evolutionary lens. NPJ Biofilms Microbiomes. 2022 Oct 18;8(1):82. 36257971 10.1038/s41522-022-00346-4 PMC9579162

[21] Uruén C, Chopo-Escuin G, Tommassen J, Mainar-Jaime RC, Arenas J. Biofilms as Promoters of Bacterial Antibiotic Resistance and Tolerance. Antibiotics. 2020 Dec 23;10(1):3. 33374551 10.3390/antibiotics10010003 PMC7822488

[22] Ahmed W, Zhai Z, Gao C. Adaptive antibacterial biomaterial surfaces and their applications. Mater Today Bio. 2019 Mar;2:100017. 10.1016/j.mtbio.2019.100017 PMC7061676 32159147

[23] Lee AS, de Lencastre H, Garau J, Kluytmans J, Malhotra-Kumar S, Peschel A, Methicillin-resistant Staphylococcus aureus. Nat Rev Dis Primers. 2018 May 31;4(1):18033. 29849094 10.1038/nrdp.2018.33

[24] Kandel CE, Jenkinson R, Daneman N, Backstein D, Hansen BE, Muller MP, Predictors of Treatment Failure for Hip and Knee Prosthetic Joint Infections in the Setting of 1- and 2-Stage Exchange Arthroplasty: A Multicenter Retrospective Cohort. Open Forum Infect Dis. 2019 Nov;6(11):ofz452. 31737739 10.1093/ofid/ofz452 PMC6847009

[25] Rodriguez-Merchan EC, Delgado-Martinez AD. Risk Factors for Periprosthetic Joint Infection after Primary Total Knee Arthroplasty. J Clin Med. 2022 Oct 18;11(20):6128. 36294449 10.3390/jcm11206128 PMC9605414

[26] Tarabichi S, Parvizi J. Prevention of surgical site infection: a ten-step approach. Arthroplasty. 2023 Apr 8;5(1):21. 37029444 10.1186/s42836-023-00174-7 PMC10082525

[27] Le Vavasseur B, Zeller V. Antibiotic Therapy for Prosthetic Joint Infections: An Overview. Antibiotics (Basel). 2022 Apr 5;11(4). 10.3390/antibiotics11040486 PMC9025623 35453237

[28] Tucci G, Romanini E, Zanoli G, Pavan L, Fantoni M, Venditti M. Prevention of surgical site infections in orthopaedic surgery: a synthesis of current recommendations. Eur Rev Med Pharmacol Sci. 2019 Apr;23(2 Suppl):224–39. 10.26355/eurrev_201904_17497 30977890

[29] Olivares E, Badel-Berchoux S, Provot C, Prévost G, Bernardi T, Jehl F. Clinical Impact of Antibiotics for the Treatment of Pseudomonas aeruginosa Biofilm Infections. Front Microbiol. 2020 Jan 9;10. 10.3389/fmicb.2019.02894 PMC6962142 31998248

[30] Wang Z, Koirala B, Hernandez Y, Zimmerman M, Park S, Perlin DS, A naturally inspired antibiotic to target multidrug-resistant pathogens. Nature. 2022 Jan;601(7894):606–11. 34987225 10.1038/s41586-021-04264-x PMC10321319

[31] Barbarossa A, Rosato A, Corbo F, Clodoveo ML, Fracchiolla G, Carrieri A, Non-Antibiotic Drug Repositioning as an Alternative Antimicrobial Approach. Antibiotics. 2022 Jun 17;11(6):816. 35740222 10.3390/antibiotics11060816 PMC9220406

[32] Bryson DJ, Morris DLJ, Shivji FS, Rollins KR, Snape S, Ollivere BJ. Antibiotic prophylaxis in orthopaedic surgery. Bone Joint J. 2016 Aug;98-B(8):1014–9. 27482011 10.1302/0301-620X.98B8.37359

[33] Gil D, Daffinee K, Friedman R, Bhushan B, Muratoglu OK, LaPlante K, Synergistic antibacterial effects of analgesics and antibiotics against Staphylococcus aureus. Diagn Microbiol Infect Dis. 2020 Apr 1;96(4):114967. 32057521 10.1016/j.diagmicrobio.2019.114967

[34] Meletiadis J, Pournaras S, Roilides E, Walsh TJ. Defining Fractional Inhibitory Concentration Index Cutoffs for Additive Interactions Based on Self-Drug Additive Combinations, Monte Carlo Simulation Analysis, and *In Vitro* - *In Vivo* Correlation Data for Antifungal Drug Combinations against *Aspergillus fumigatus*. Antimicrob Agents Chemother. 2010 Feb;54(2):602–9. 19995928 10.1128/AAC.00999-09 PMC2812160

[35] Te Dorsthorst DTA, Verweij PE, Meis JFGM, Punt NC, Mouton JW. Comparison of Fractional Inhibitory Concentration Index with Response Surface Modeling for Characterization of In Vitro Interaction of Antifungals against Itraconazole-Susceptible and -Resistant *Aspergillus fumigatus* Isolates. Antimicrob Agents Chemother. 2002 Mar;46(3):702–7. 11850251 10.1128/AAC.46.3.702-707.2002 PMC127491

[36] Mountcastle SE, Vyas N, Villapun VM, Cox SC, Jabbari S, Sammons RL, Biofilm viability checker: An open-source tool for automated biofilm viability analysis from confocal microscopy images. NPJ Biofilms Microbiomes. 2021 May 14;7(1):44. 33990612 10.1038/s41522-021-00214-7 PMC8121819

[37] Bossù M, Selan L, Artini M, Relucenti M, Familiari G, Papa R, Characterization of Scardovia wiggsiae Biofilm by Original Scanning Electron Microscopy Protocol. Microorganisms. 2020 May 27;8(6):807. 32471210 10.3390/microorganisms8060807 PMC7355790

[38] Livak KJ, Schmittgen TD. Analysis of Relative Gene Expression Data Using Real-Time Quantitative PCR and the 2−ΔΔCT Method. Methods. 2001 Dec;25(4):402–8. 11846609 10.1006/meth.2001.1262

[39] Wenzel M, Vischer N, Strahl H, Hamoen L. Assessing Membrane Fluidity and Visualizing Fluid Membrane Domains in Bacteria Using Fluorescent Membrane Dyes. Bio Protoc. 2018;8(20). 10.21769/BioProtoc.3063 PMC8342135 34532528

[40] Peng J, Mishra B, Khader R, Felix L, Mylonakis E. Novel Cecropin-4 Derived Peptides against Methicillin-Resistant Staphylococcus aureus. Antibiotics. 2021 Jan 1;10(1):36. 33401476 10.3390/antibiotics10010036 PMC7824259

[41] Mandell JB, Orr S, Koch J, Nourie B, Ma D, Bonar DD, Large variations in clinical antibiotic activity against Staphylococcus aureus biofilms of periprosthetic joint infection isolates. J Orthop Res. 2019 Jul;37(7):1604–9. 30919513 10.1002/jor.24291 PMC7141781

[42] Mahmoodi AN, Kim PY. Ketorolac. 2023. 31424756

[43] Dalury DF. A state-of-the-art pain protocol for total knee replacement. Arthroplast Today. 2016 Mar;2(1):23–5. 28326393 10.1016/j.artd.2016.01.004 PMC4957167

[44] Loss G, Simões PM, Valour F, Cortês MF, Gonzaga L, Bergot M, Staphylococcus aureus Small Colony Variants (SCVs): News From a Chronic Prosthetic Joint Infection. Front Cell Infect Microbiol. 2019 Oct 22;9. 10.3389/fcimb.2019.00363 PMC6817495 31696062

[45] Paharik AE, Horswill AR. The Staphylococcal Biofilm: Adhesins, Regulation, and Host Response. Microbiol Spectr. 2016 Mar 25;4(2). 10.1128/microbiolspec.VMBF-0022-2015 PMC4887152 27227309

[46] Epand RM, Walker C, Epand RF, Magarvey NA. Molecular mechanisms of membrane targeting antibiotics. Biochim Biophys Acta. 2016 May;1858(5):980–7. 26514603 10.1016/j.bbamem.2015.10.018

[47] Kim W, Zou G, Hari TPA, Wilt IK, Zhu W, Galle N, A selective membrane-targeting repurposed antibiotic with activity against persistent methicillin-resistant Staphylococcus aureus. Proc Natl Acad Sci U S A. 2019 Aug 13;116(33):16529–34. 31358625 10.1073/pnas.1904700116 PMC6697817

[48] Dombach JL, Quintana JLJ, Nagy TA, Wan C, Crooks AL, Yu H, A small molecule that mitigates bacterial infection disrupts Gram-negative cell membranes and is inhibited by cholesterol and neutral lipids. PLoS Pathog. 2020 Dec;16(12):e1009119. 33290418 10.1371/journal.ppat.1009119 PMC7748285

[49] Nguyen TLA, Bhattacharya D. Antimicrobial Activity of Quercetin: An Approach to Its Mechanistic Principle. Molecules. 2022 Apr 12;27(8):2494. 35458691 10.3390/molecules27082494 PMC9029217

[50] Tam VH, Kabbara S, Vo G, Schilling AN, Coyle EA. Comparative Pharmacodynamics of Gentamicin against *Staphylococcus aureus* and *Pseudomonas aeruginosa*. Antimicrob Agents Chemother. 2006 Aug;50(8):2626–31. 16870751 10.1128/AAC.01165-05 PMC1538660

[51] Schafer JA. Consistent rates of kill of Staphylococcus aureus by gentamicin over a 6-fold clinical concentration range in an in vitro pharmacodynamic model (IVPDM). Journal of Antimicrobial Chemotherapy. 2006 May 2;58(1):108–11. 16735429 10.1093/jac/dkl216

[52] Steadman W, Chapman PR, Schuetz M, Schmutz B, Trampuz A, Tetsworth K. Local Antibiotic Delivery Options in Prosthetic Joint Infection. Antibiotics. 2023 Apr 14;12(4):752. 37107114 10.3390/antibiotics12040752 PMC10134995

[53] Sreedharan Nair V, Ganeshan Radhamony N, Rajendra R, Mishra R. Effectiveness of intraoperative periarticular cocktail injection for pain control and knee motion recovery after total knee replacement. Arthroplast Today. 2019 Sep;5(3):320–4. 31516975 10.1016/j.artd.2019.05.004 PMC6728801

[54] Bernthal NM, Hart CM, Sheth KR, Bergese SD, Ho HS, Apfel CC, Local and Intra-articular Administration of Nonsteroidal Anti-inflammatory Drugs for Pain Management in Orthopedic Surgery. Am J Ther. 2022 Mar;29(2):e219–28. 10.1097/MJT.0000000000001309 33315593

[55] Li JW, Ma YS, Xiao LK. Postoperative Pain Management in Total Knee Arthroplasty. Orthop Surg. 2019 Oct;11(5):755–61. 31663286 10.1111/os.12535 PMC6819170

[56] Macià MD, Rojo-Molinero E, Oliver A. Antimicrobial susceptibility testing in biofilm-growing bacteria. Clin Microbiol Infect. 2014 Oct;20(10):981–90. 24766583 10.1111/1469-0691.12651

[57] Eliopoulos GM, Moellering RC. Antibiotic synergism and antimicrobial combinations in clinical infections. Rev Infect Dis. 1982;4(2):282–93. 7051231 10.1093/clinids/4.2.282

[58] Ejim L, Farha MA, Falconer SB, Wildenhain J, Coombes BK, Tyers M, Combinations of antibiotics and nonantibiotic drugs enhance antimicrobial efficacy. Nat Chem Biol. 2011 Jun 24;7(6):348–50. 21516114 10.1038/nchembio.559

[59] Manohar P, Madurantakam Royam M, Loh B, Bozdogan B, Nachimuthu R, Leptihn S. Synergistic Effects of Phage–Antibiotic Combinations against *Citrobacter amalonaticus*. ACS Infect Dis. 2022 Jan 14;8(1):59–65. 34979073 10.1021/acsinfecdis.1c00117

[60] Duong L, Gross SP, Siryaporn A. Developing Antimicrobial Synergy With AMPs. Front Med Technol. 2021 Mar 12;3. 10.3389/fmedt.2021.640981 PMC8757689 35047912

[61] Sedlmayer F, Woischnig AK, Unterreiner V, Fuchs F, Baeschlin D, Khanna N, 5-Fluorouracil blocks quorum-sensing of biofilm-embedded methicillin-resistant *Staphylococcus aureus* in mice. Nucleic Acids Res. 2021 Jul 21;49(13):e73–e73. 33856484 10.1093/nar/gkab251 PMC8287944

[62] Cella MA, Coulson T, MacEachern S, Badr S, Ahmadi A, Tabatabaei MS, Probiotic disruption of quorum sensing reduces virulence and increases cefoxitin sensitivity in methicillin-resistant Staphylococcus aureus. Sci Rep. 2023 Mar 16;13(1):4373. 36928453 10.1038/s41598-023-31474-2 PMC10020441

[63] Kim W, Zou G, Hari TPA, Wilt IK, Zhu W, Galle N, A selective membrane-targeting repurposed antibiotic with activity against persistent methicillin-resistant *Staphylococcus aureus*. Proceedings of the National Academy of Sciences. 2019 Aug 13;116(33):16529–34. 10.1073/pnas.1904700116 PMC6697817 31358625

[64] Kamurai B, Mombeshora M, Mukanganyama S. Repurposing of Drugs for Antibacterial Activities on Selected ESKAPE Bacteria Staphylococcus aureus and Pseudomonas aeruginosa. Int J Microbiol. 2020;2020:8885338. 33061985 10.1155/2020/8885338 PMC7542517

[65] Schneider EK, Reyes-Ortega F, Velkov T, Li J. Antibiotic–non-antibiotic combinations for combating extremely drug-resistant Gram-negative ‘superbugs.’ Essays Biochem. 2017 Mar 3;61(1):115–25. 28258235 10.1042/EBC20160058

[66] Kim HS, Park JW, Moon SY, Lee YK, Ha YC, Koo KH. Current and Future Burden of Periprosthetic Joint Infection from National Claim Database. J Korean Med Sci. 2020 Dec 21;35(49):e410. 33350183 10.3346/jkms.2020.35.e410 PMC7752258

[67] Patel R. Periprosthetic Joint Infection. New England Journal of Medicine. 2023 Jan 19;388(3):251–62. 36652356 10.1056/NEJMra2203477

[68] Gardete S, Wu SW, Gill S, Tomasz A. Role of VraSR in Antibiotic Resistance and Antibiotic-Induced Stress Response in *Staphylococcus aureus*. Antimicrob Agents Chemother. 2006 Oct;50(10):3424–34. 17005825 10.1128/AAC.00356-06 PMC1610096

[69] Kuroda M, Kuroda H, Oshima T, Takeuchi F, Mori H, Hiramatsu K. Two-component system VraSR positively modulates the regulation of cell-wall biosynthesis pathway in Staphylococcus aureus. Mol Microbiol. 2004 Jan 28;49(3):807–21. 10.1046/j.1365-2958.2003.03599.x 12864861

[70] Galbusera E, Renzoni A, Andrey DO, Monod A, Barras C, Tortora P, Site-Specific Mutation of *Staphylococcus aureus* VraS Reveals a Crucial Role for the VraR-VraS Sensor in the Emergence of Glycopeptide Resistance. Antimicrob Agents Chemother. 2011 Mar;55(3):1008–20. 21173175 10.1128/AAC.00720-10 PMC3067069

[71] Proctor RA, Kriegeskorte A, Kahl BC, Becker K, LÃ¶ffler B, Peters G. Staphylococcus aureus Small Colony Variants (SCVs): a road map for the metabolic pathways involved in persistent infections. Front Cell Infect Microbiol. 2014 Jul 28;4. 10.3389/fcimb.2014.00099 PMC4112797 25120957

[72] Tande AJ, Osmon DR, Greenwood-Quaintance KE, Mabry TM, Hanssen AD, Patel R. Clinical Characteristics and Outcomes of Prosthetic Joint Infection Caused by Small Colony Variant Staphylococci. mBio. 2014 Oct 31;5(5). 10.1128/mBio.01910-14 PMC4196237 25271290

[73] Park PW, Rosenbloom J, Abrams WR, Rosenbloom J, Mecham RP. Molecular Cloning and Expression of the Gene for Elastin-binding Protein (ebpS) in Staphylococcus aureus. Journal of Biological Chemistry. 1996 Jun;271(26):15803–9. 8663124 10.1074/jbc.271.26.15803

[74] Hudson MC, Ramp WK, Frankenburg KP. *Staphylococcus aureus* adhesion to bone matrix and bone-associated biomaterials. FEMS Microbiol Lett. 1999 Apr;173(2):279–84. 10227156 10.1111/j.1574-6968.1999.tb13514.x

[75] Pizauro LJL, de Almeida CC, Silva SR, MacInnes JI, Kropinski AM, Zafalon LF, Genomic comparisons and phylogenetic analysis of mastitis-related staphylococci with a focus on adhesion, biofilm, and related regulatory genes. Sci Rep. 2021 Aug 30;11(1):17392. 34462461 10.1038/s41598-021-96842-2 PMC8405628

[76] O’Gara JP. *ica* and beyond: biofilm mechanisms and regulation in *Staphylococcus epidermidis* and *Staphylococcus aureus*. FEMS Microbiol Lett. 2007 May;270(2):179–88. 17419768 10.1111/j.1574-6968.2007.00688.x

[77] Fey PD, Olson ME. Current concepts in biofilm formation of *Staphylococcus epidermidis*. Future Microbiol. 2010 Jun;5(6):917–33. 20521936 10.2217/fmb.10.56 PMC2903046

[78] Olson ME, Garvin KL, Fey PD, Rupp ME. Adherence of Staphylococcus epidermidis to Biomaterials Is Augmented by PIA. Clin Orthop Relat Res. 2006 Oct;451:21–4. 16906069 10.1097/01.blo.0000229320.45416.0c

[79] Hess DJ, Henry-Stanley MJ, Wells CL. Gentamicin Promotes Staphylococcus aureus Biofilms on Silk Suture. Journal of Surgical Research. 2011 Oct;170(2):302–8. 21816417 10.1016/j.jss.2011.06.011 PMC3246803

[80] Arciola CR, Campoccia D, Ravaioli S, Montanaro L. Polysaccharide intercellular adhesin in biofilm: structural and regulatory aspects. Front Cell Infect Microbiol. 2015 Feb 10;5. 10.3389/fcimb.2015.00007 PMC4322838 25713785

[81] Yoon Y, Lee H, Lee S, Kim S, Choi KH. Membrane fluidity-related adaptive response mechanisms of foodborne bacterial pathogens under environmental stresses. Food Research International. 2015 Jun 1;72:25–36.

[82] Sanchez SA, Tricerri MA, Gratton E. Laurdan generalized polarization fluctuations measures membrane packing micro-heterogeneity in vivo. Proceedings of the National Academy of Sciences. 2012 May 8;109(19):7314–9. 10.1073/pnas.1118288109 PMC3358851 22529342

[83] Radlinski LC, Rowe SE, Brzozowski R, Wilkinson AD, Huang R, Eswara P, Chemical Induction of Aminoglycoside Uptake Overcomes Antibiotic Tolerance and Resistance in Staphylococcus aureus. Cell Chem Biol. 2019 Oct;26(10):1355–1364.e4. 31402316 10.1016/j.chembiol.2019.07.009 PMC6800641

[84] Yarlagadda V, Wright GD. Membrane-Active Rhamnolipids Overcome Aminoglycoside Resistance. Cell Chem Biol. 2019 Oct;26(10):1333–4. 31626780 10.1016/j.chembiol.2019.09.015

[85] Allison KR, Brynildsen MP, Collins JJ. Metabolite-enabled eradication of bacterial persisters by aminoglycosides. Nature. 2011 May 11;473(7346):216–20. 21562562 10.1038/nature10069 PMC3145328

[86] Müller A, Wenzel M, Strahl H, Grein F, Saaki TN V., Kohl B, Daptomycin inhibits cell envelope synthesis by interfering with fluid membrane microdomains. Proceedings of the National Academy of Sciences. 2016 Nov 8;113(45). 10.1073/pnas.1611173113 PMC5111643 27791134

[87] Epand RM, Epand RF. Lipid domains in bacterial membranes and the action of antimicrobial agents. Biochimica et Biophysica Acta (BBA) - Biomembranes. 2009 Jan;1788(1):289–94. 18822270 10.1016/j.bbamem.2008.08.023

[88] Suhardi VJ, Bichara DA, Kwok SJJ, Freiberg AA, Rubash H, Malchau H, A fully functional drug-eluting joint implant. Nat Biomed Eng. 2017 Jun 13;1(6):0080. 29354321 10.1038/s41551-017-0080 PMC5773111

[89] Gil D, Hugard S, Borodinov N, Ovchinnikova OS, Muratoglu OK, Bedair H, Dual-analgesic loaded UHMWPE exhibits synergistic antibacterial effects against *Staphylococci*. J Biomed Mater Res B Appl Biomater. 2023 Apr 3;111(4):912–22. 36462210 10.1002/jbm.b.35201

[90] Gil D, Atici AE, Connolly RL, Hugard S, Shuvaev S, Wannomae KK, Addressing prosthetic joint infections via gentamicin-eluting UHMWPE spacer. Bone Joint J. 2020 Jun;102-B(6_Supple_A):151–7. 32475290 10.1302/0301-620X.102B6.BJJ-2019-1593.R1

[91] Grindy S, Gil D, Suhardi J, Fan Y, Moore K, Hugard S, Hydrogel device for analgesic drugs with in-situ loading and polymerization. Journal of Controlled Release. 2023 Sep 1;361:20–8. 37451545 10.1016/j.jconrel.2023.07.022

